# Correlation of CSF flow using phase-contrast MRI with ventriculomegaly and CSF opening pressure in mucopolysaccharidoses

**DOI:** 10.1186/s12987-017-0073-2

**Published:** 2017-09-18

**Authors:** Amauri Dalla Corte, Carolina F. M. de Souza, Maurício Anés, Fabio K. Maeda, Armelle Lokossou, Leonardo M. Vedolin, Maria Gabriela Longo, Monica M. Ferreira, Solanger G. P. Perrone, Olivier Balédent, Roberto Giugliani

**Affiliations:** 1 Post-Graduate Program in Medical Sciences, Universidade Federal do Rio Grande do Sul, Porto Alegre, Brazil; 2 Medical Genetics Service, Hospital de Clínicas de Porto Alegre, Rua Ramiro Barcelos 2350, Porto Alegre, RS 90035-903 Brazil; 3 Medical Physics and Radioprotection Service, Hospital de Clínicas de Porto Alegre, Porto Alegre, Brazil; 4 Clinical Engineering, Santa Casa de Misericórdia de Porto Alegre, Porto Alegre, Brazil; 5 Image Processing Unit, Amiens University Hospital, Amiens, France; 6 Department of Neuroradiology, DASA Group, São Paulo, Brazil; 7 Department of Radiology, Massachusetts General Hospital, Boston, USA; 8 Anesthesiology Service, Hospital de Clínicas de Porto Alegre, Porto Alegre, Brazil

**Keywords:** Mucopolysaccharidoses, Brain MRI, Ventricular enlargement, Hydrocephalus, Cerebrospinal fluid

## Abstract

**Background:**

Very little is known about the incidence and prevalence of hydrocephalus in patients with mucopolysaccharidoses (MPS). The biggest challenge is to distinguish communicating hydrocephalus from ventricular dilatation secondary to brain atrophy, because both conditions share common clinical and neuroradiological features. The main purpose of this study is to assess the relationship between ventriculomegaly, brain and cerebrospinal fluid (CSF) volumes, aqueductal and cervical CSF flows, and CSF opening pressure in MPS patients, and to provide potential biomarkers for abnormal CSF circulation.

**Methods:**

Forty-three MPS patients (12 MPS I, 15 MPS II, 5 MPS III, 9 MPS IV A and 2 MPS VI) performed clinical and developmental tests, and T1, T2, FLAIR and phase-contrast magnetic resonance imaging (MRI) followed by a lumbar puncture with the CSF opening pressure assessment. For the analysis of MRI variables, we measured the brain and CSF volumes, white matter (WM) lesion load, Evans’ index, third ventricle width, callosal angle, dilated perivascular spaces (PVS), craniocervical junction stenosis, aqueductal and cervical CSF stroke volumes, and CSF glycosaminoglycans concentration.

**Results:**

All the scores used to assess the supratentorial ventricles enlargement and the ventricular CSF volume presented a moderate correlation with the aqueductal CSF stroke volume (ACSV). The CSF opening pressure did not correlate either with the three measures of ventriculomegaly, or the ventricular CSF volume, or with the ACSV. Dilated PVS showed a significant association with the ventriculomegaly, ventricular CSF volume and elevated ACSV.

**Conclusions:**

In MPS patients ventriculomegaly is associated with a severe phenotype, increased cognitive decline, WM lesion severity and enlarged PVS. The authors have shown that there are associations between CSF flow measurements and measurements related to CSF volumetrics. There was also an association of volumetric measurements with the degree of dilated PVS.

## Background

The mucopolysaccharidoses (MPS) are a group of rare genetic disorders of glycosaminoglycan (GAG) catabolism. Each MPS disorder is caused by a deficiency in the activity of a single, specific lysosomal enzyme required for GAG degradation. Lysosomal accumulation of GAGs results in chronic and progressive cellular damage, which can affect multiple organ systems. The neurologic expression of the disease varies among the different MPS types and sometimes also within the same type [1]. Ventricular enlargement is known to occur in patients with MPS and may be due to the combination of cortical atrophy secondary to central nervous system degeneration, a defect in cerebrospinal fluid (CSF) reabsorption due to thickening of the meninges and dysfunction of the Pacchionian granulations in the arachnoid villi, and venous hypertension secondary to reduced venous outflow through bone dysostosis of the skull base [2, 3]. The communicating hydrocephalus that occurs in MPS is usually slowly progressive and difficult to distinguish from the primary neurologic disease. Acute symptoms such as vomiting and papilledema are uncommon. The ventricular enlargement in severe forms of MPS I (Hurler syndrome) and MPS II (Hunter syndrome) may be associated with increased intracranial pressure (ICP), which can be used as indication for a shunting procedure. The degree to which hydrocephalus contributes to the neurologic deterioration in MPS is unknown [1].

Because brain atrophy and communicating hydrocephalus share common clinical and neuroradiological features in MPS patients, and in addition, they can potentially coexist, these two conditions were previously considered as one [4]. Ventriculomegaly is a cardinal feature of hydrocephalus and the severity can be defined by an Evans’ index greater than 0.3 [5]. Several authors used magnetic resonance imaging (MRI) scoring systems to grade the ventricular enlargement and help differentiate between hydrocephalus and ex vacuo ventriculomegaly in MPS patients, taking into account mainly the width of the third ventricle and temporal horn dilatation [2, 6–9]. Measuring the callosal angle has been suggested as a convenient method for discriminating idiopathic normal pressure hydrocephalus (INPH) from neurodegenerative disease with large ventricles due to atrophy [10]. Excess build-up of CSF leads to an increase in ICP, which can be equated to CSF opening pressure. The relationship between size of cerebral ventricles and ICP has previously been investigated in adults and children with hydrocephalus, and conventional knowledge says that, with exception of some acute cases, brain imaging is rarely helpful in guessing value of ICP quantitatively [11–14]. We recently published an algorithm to aid in the diagnosis and management of hydrocephalus in MPS patients, considering neurologic deterioration, Evans’ index, width of the third ventricle, callosal angle, CSF opening pressure and the presence of craniocervical stenosis [15].

At present, neuroimaging has only been only used to verify the extent of ventriculomegaly and exclude cases of gross cerebral atrophy and other pathological conditions that might explain the neurological deterioration in MPS patients. Phase-contrast (PC) MRI provides valuable additional information to conventional MRI and could help to distinguish hydrocephalus from brain atrophy in MPS patients by measuring the volume of CSF pulsating back and forth through the aqueduct with each cardiac cycle, the aqueductal CSF stroke volume (ACSV). According to Bradley, hyperdynamic CSF flow through the aqueduct is seen when there is ventricular enlargement without cerebral atrophy [16]. However, while some studies have supported the use of aqueductal flow rate for the diagnosis of INPH [17, 18] and related flow rate to a possible shunt response [19, 20], other studies have not been able to demonstrate any association between a clinical improvement after shunting and increased ACSV [21–23] or flow rate [24].

Currently, there is a lack of consensus as to which diagnostic test most reliably predicts which MPS patients will benefit from CSF diversion. Moreover, it has proved to be very difficult, using any objective measure, to demonstrate a neurological improvement after CSF drainage that could indicate an eventual positive response to ventriculoperitoneal shunting (VPS) [25]. There is also great difficulty in investigating these patients due to: rare disease, behavioral disturbances, high anesthetic risk, technical difficulties for lumbar puncture and low CSF drainage, for which invasive tests such as intermittent high volume spinal tap, prolonged lumbar drainage and continuous ICP monitoring are not feasible [15]. In this context, a noninvasive tool for selecting MPS patients with hydrocephalus for surgery would certainly be preferable, if the evidence for its utility was convincing.

The purpose of this study was to provide images of both structural and quantitative changes in the brain allowing access to and a better understanding of the brain disease and the consequences of hydrocephalus in patients with MPS. In that sense, we aimed to be able to identify noninvasive potential biomarkers for abnormal CSF circulation. Besides that, we analyzed the relationship between ventricular and cervical CSF flows and ventricular size, brain and CSF volumes, CSF opening pressure, CSF GAGs levels, dilated perivascular spaces (PVS), white matter (WM) lesion load, and craniocervical junction stenosis.

## Methods

### Subjects

This was a cross-sectional study. From July 2013 to June 2016, we examined all patients with MPS followed at the Medical Genetics Service of Hospital de Clínicas de Porto Alegre. Of these, 47 patients with confirmed biochemical diagnosis of MPS underwent evaluation for ventriculomegaly. We excluded three subjects who had VPS and one who was unable to undergo the MRI due to respiratory compromise and clinical instability. The remaining 43 patients formed the study group (12 patients with MPS I, 15 with MPS II, 5 with MPS III, 9 with MPS IV A, and 2 with MPS VI). Of the MPS I patients, 5 had Hurler syndrome, 2 had Hurler-Scheie syndrome and 5 had Scheie syndrome. Of the MPS II patients, 8 had the severe form and 7 had the attenuated form. Of the MPS III patients, 1 had type A, 3 had type B and 1 had type C. Twenty-five patients were male and 18 were female. Each patient presented with typical clinical manifestations of the disorder and had biochemical confirmation of a deficient enzymatic activity (α-l-iduronidase for MPS I, iduronate sulfatase for MPS II, heparan *N*-sulfatase for MPS III A, α-*N*-acetyl-glucosaminidase for MPS III B, acetyl-CoA: α-glucosaminide acetyltransferase for MPS III C, galactose 6-sulfatase for MPS IVA, and *N*-acetylgalactosamine 4-sulfatase for MPS VI). Multiple sulfatase deficiency was excluded by the observation of a normal activity of at least one other sulfatase. Twenty patients were receiving enzyme replacement therapy (7 with MPS I, 10 with MPS II, 1 with MPS IV A and 2 with MPS VI). Each patient had brain MR imaging immediately followed by lumbar puncture, and neurodevelopmental assessment performed within the same week, except one patient who died before intellectual test could be applied.

### Neurodevelopmental assessment

All patients received age-appropriate standardized neurodevelopmental assessments: Bayley Scales of Infant and Toddler Development Third Edition (Bayley-III) for children younger than 42 months, Wechsler Preschool and Primary Scale of Intelligence—Revised (WPPSI-R) for children between 42 months and 6 years, Wechsler Intelligence Scale for Children Third Edition (WISC-III) for patients between 6 and 16 years, and Wechsler Adult Intelligence Scale Third Edition (WAIS-III) for patients who were 16 years or older. All tests were performed according to their guidelines and to the developmental level of each patient by a psychologist (S.G.P.P.) who was experienced in development neurology. According to the study protocol, full-scale IQ scores were rated and patients presented as having cognitive impairment (CI) or not. CI was considered present when developmental tests (composite score) or intelligence quotient (IQ) <70. Severely affected patients who could not respond to development tests were classified within the CI group.

### CSF sampling

Each patient underwent a lumbar puncture in a flexed lateral decubitus position whereby 7–10 cc of CSF was removed. The CSF opening pressure was measured with a standard spinal manometer calibrated in mm H_2_O (Hako, Germany) connected to a 20-G spinal needle. In five patients, it was not possible to obtain the opening pressure or the CSF sample due to technical difficulties (3 patients) or refusal to undergo the procedure after performing MRI (2 patients).

### Data acquisition

MRI studies were obtained on 1.5T Achieva (Philips Medical Systems, Best, The Netherlands) software version 2.6.3. For the acquisition of the cerebral images and the cervical region we used a 16-channel neurovascular coil manufactured by Invivo Devices. The research protocol for brain images included *fast spin echo* transversal plane FLAIR (TR 11000 ms; TE 140 ms; IT 2800 ms; flip 90°; NSA 3; slice thickness 5 mm; gap 1 mm; matrix 169 × 225; in plane resolution 1.3 × 1.03 mm; echo train length 55); *fast spin echo* transversal plane T2 (5054 ms; 100 ms; 90°; 2; 5 mm; 1 mm; 219 × 292; 0.75 × 0.59; 15); MPRAGE sagittal plane T1 (8.69 ms; 4 ms; 8°; 1 mm; 232 × 256; 1 × 1 mm; 232); cervical spine included *fast spin echo* T2 sagittal plane (4735 ms; 100 ms; 90°; 4; 3 mm; 0.5 mm; 247 × 198; 1 × 0.9 mm; 24); *fast spin echo* T2 axial plane (3800 ms; 120 ms; 90°; 4; 3 mm; 0.4 mm; 247 × 198; 1.11 × 0.85 mm; 44); *fast spin echo* T1 sagittal plane (958 ms; 7.8 ms; 90°; 3; 3 mm; 0.5 mm; 247 × 198; 1.25 × 0.9 mm; 4); throughout plane flow was measured with PC gradient echo for CSF at aqueduct and cervical subarachnoid space at C2–C3 spine level with velocity encoding (Venc) equal to 12 cm/s (TR 21 ms; TE 12 ms; flip 10°; NSA 2; slice thickness 5 mm; no gap; matrix 182 × 182; in plane resolution 0.55 × 0.55 mm) and 10 cm/s (21 ms; 12 ms; 10°; 2; 5 mm; 220 × 182; 0.55 × 0.5 mm), respectively. For those patients whose CSF flow was very distinct, the Venc was adjusted accordingly to reduce flow void artifacts and achieve higher image contrast.

### Imaging processing

Neuroimage post-processing was performed at a workstation by three researchers in agreement (A.D.C., M.A. and F.K.M.). They were blinded to the age, type, and clinical status of the patients.

#### Lesion load, CSF and brain volumes

Cerebral segmentation and WM lesion load were measured with FLAIR brain images and CSF with T2-weighted images. The segmentation process and volume quantification were performed as described in detail elsewhere [26] to obtain brain volume, total CSF volume, ventricular CSF volume, subarachnoid CSF volume and lesion load. The skull size was used as denominator to correct brain and CSF volumes for variations in head size.

#### Size of the supratentorial ventricles

Evans’ index was calculated as the ratio of the greatest width of the frontal horns of the lateral ventricles to the maximal internal diameter of the skull [5]. The third ventricle enlargement was graded as follows: 1 = width of the third ventricle <5 mm; 2 = width of the third ventricle between 5 and 10 mm; 3 = width of the third ventricle >10 mm [9]. The callosal angle was measured on the coronal plane, which was perpendicular to the anteroposterior commissure plane on the posterior commissure of each subject [10]. The Evans’ index, the width of third ventricle and the corpus callosal angle were calculated on the individual MPRAGE sagittal T1 reoriented to the parallel plane from the anteroposterior commissure plane consisting of 1.0-mm isotropic voxels. Ventriculomegaly was defined as Evans’ index >0.3, width of the third ventricle >10 mm, or callosal angle <90°.

#### Enlargement of the perivascular spaces

The PVS enlargement on T1-weighted images located in periventricular and subcortical WM, corpus callosum, basal ganglia, thalami and brainstem, were graded as follows: 0 = none; 1 = PVS number <10 and PVS size <3 mm; 2 = PVS number ≥10 and PVS size <3 mm; 3 = PVS number ≥10 and PVS size ≥3 mm [9].

#### Craniocervical junction stenosis

For MRI evaluation of the craniocervical junction, sagittal and axial T1- and T2-weighted images were created. The T2-weighted images were also examined for increased signal intensity, suggesting myelomalacia. The presence or absence of a compression of the spinal cord was graded as follows: 0 = no spinal cord compression; 1 = spinal cord compression (absence of CSF in any direction); 2 = signs of myelomalacia [27].

#### Aqueductal and cervical CSF stroke volume

**Fig. 1:**
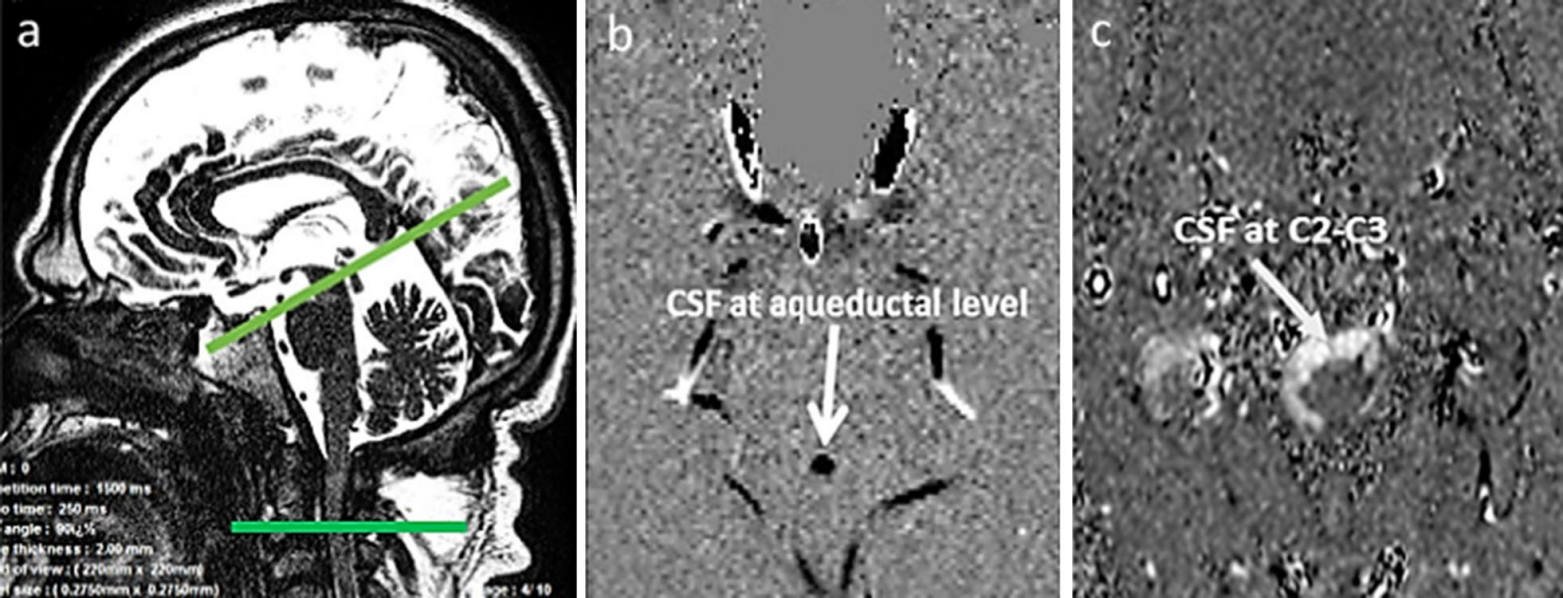
Data acquisition of a 34-year-old male patient with MPS II. Sagittal 3D scout view sequences were used as localizer to select the anatomical levels for flow quantification (**a**). The acquisition planes were selected perpendicular to the presumed direction of the flows. Sections through the cerebral aqueduct (**b**) and C2–C3 subarachnoid space level (**c**) were used for CSF flow measurement

**Fig. 2:**
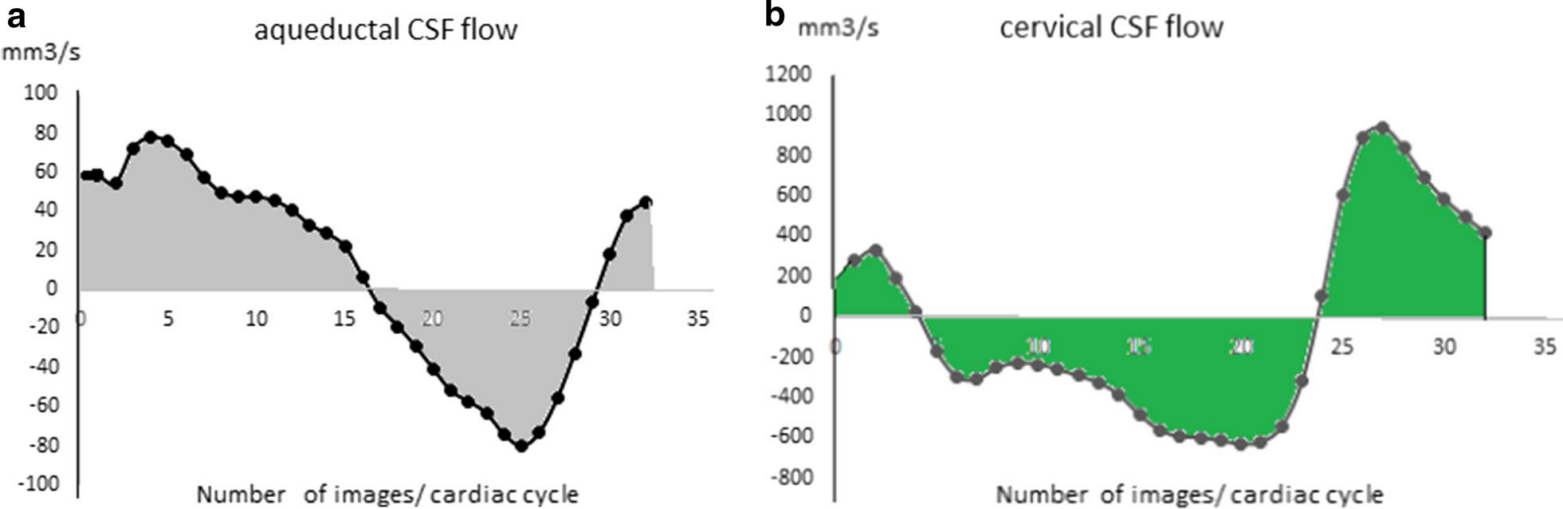
CSF oscillations were reconstructed during the cardiac cycle at aqueductal level (**a**) and cervical level (**b**). Aqueductal CSF stroke volume and cervical CSF stroke volume represent the mean volume of CSF under the curve of CSF flow during the cardiac cycle

Stroke volumes were defined as the average of craniocaudal and caudocranial volumes displaced through the region of interest (ROI) during the cardiac cycle (CC) [28]. They were measured for CSF in the aqueduct and cervical level and were expressed in milliliters per CC. Data were analyzed using validated image processing software [29] with an optimized CSF flow segmentation algorithm, which automatically extracts the ROI at each level, and calculates its flow curves over the 32 segments of the CC. Then, the CSF flow curve was generated within one CC (Figs. 1, 2). High CSF stroke volume was defined as aqueductal >0.05 ml/CC and C2–C3 >0.5 ml/CC [30].

### CSF GAGs analysis

Total CSF GAGs concentration was determined using a thrombin activity assay. The CSF samples were preincubated with human heparin cofactor II (HC II) and then incubated with a fixed amount of thrombin and with 0.5 mmol/l chromogenic substrate S-2238 in assay buffer. The quantification of the GAG concentrations was performed as described elsewhere [31].

### Statistical analysis

Continuous variables were described using median and range, due to asymmetric distributions. Categorical data were presented as counts and percentages. Comparison of groups was conducted using Mann–Whitney U test or Fisher exact test, accordingly. To evaluate correlations between continuous and ordinal variables we used Spearman’s rank correlation coefficient (r_s_). Due to multiple comparisons, all p values in Table 1 were adjusted using Finner’s adjustment procedure. Additionally, odds ratios were computed to estimate magnitude of association between categorical data. In situations where the odds ratio was undefined using traditional methods we approximated using Peto odds ratio.

**Table 1:** Correlation between ventricular and CSF volume measurements, and CSF opening pressure, CSF flow and neuroimaging findings

	CSF OP			ACSV			C2–C3 CSF SV			Dilated PVS			WML load			CCJ stenosis		
	*r*_*s*_	*p*	*p*′	*r*_*s*_	*p*	*p*′	*r*_*s*_	*p*	*p*′	*r*_*s*_	*p*	*p*′	*r*_*s*_	*p*	*p*′	*r*_*s*_	*p*	*p*′
Evans’ index	−0.08	0.646	0.702	0.35	*0.023*	*0.040*	−0.05	0.762	0.813	0.39	*0.011*	*0.019*	0.51	*0.001*	*0.004*	−0.01	0.933	0.933
TVW	−0.29	0.073	0.412	0.46	*0.002*	*0.014*	−0.12	0.469	0.773	0.54	*<0.001*	*0.003*	0.34	*0.030*	0.052	−0.08	0.619	0.730
Callosal angle	0.06	0.721	0.721	−0.46	*0.002*	*0.014*	−0.20	0.204	0.773	−0.35	*0.020*	*0.028*	−0.54	*<0.001*	*0.003*	0.18	0.262	0.508
Brain volume	0.27	0.113	0.412	−0.13	0.418	0.468	0.21	0.191	0.773	−0.33	*0.033*	*0.038*	−0.01	0.975	0.975	0.13	0.426	0.622
TCSF volume	−0.18	0.300	0.460	0.29	0.071	0.098	0.09	0.596	0.773	0.49	*0.001*	*0.004*	0.26	0.097	0.133	−0.30	0.054	0.177
VCSF volume	−0.10	0.577	0.700	0.46	*0.002*	*0.014*	0.01	0.941	0.941	0.55	*<0.001*	*0.003*	0.51	*0.001*	*0.004*	−0.08	0.607	0.730
SACSF volume	−0.26	0.124	0.412	0.06	0.694	0.694	0.16	0.325	0.773	0.29	0.071	0.071	0.03	0.871	0.908	−0.37	*0.016*	0.107

Data are presented as Spearman’s rank correlation coefficient (r_s_), *p* value and adjusted *p* (*p*′) using Finner’s adjustment procedure

Italic values indicate significance of *p* value (*p* < 0.05)

*CSF* cerebrospinal fluid, *CSF OP* cerebrospinal fluid opening pressure, *ACSV* aqueductal cerebrospinal fluid stroke volume, *C2–C3 CSF SV* C2–C3 cerebrospinal fluid stroke volume, *PVS* perivascular spaces, *WML* white matter lesion, *CCJ* craniocervical junction, *TVW* third ventricle width, *TCSF* total cerebrospinal fluid, *VCSF* ventricular cerebrospinal fluid, *SACSF* subarachnoid cerebrospinal fluid

Selected continuous measurements with no prior defined cut-off points were dichotomised using median values which are presented in Table 2. Therefore, adopted cut-off values were: CSF protein level >32 mg/dl, CSF GAGs >250 ng/ml, PVS ≥10; WM lesion load >0.4 cm^3^; ACSV >0.05 ml/CC; C2–C3 CSF stroke volume >0.5 ml/CC.

**Table 2:** Association between ventriculomegaly and cerebrospinal fluid flow with clinical, cerebrospinal fluid and neuroimaging characteristics

Characteristics	Ventriculomegaly									CSF stroke volume					
	Evans’ index			Third ventricle width (mm)			Callosal angle (°)			Aqueductal (ml/CC)			C2–C3 (ml/CC)		
	>0.3 n = 15	≤0.3 n = 28	*p*	>10 n = 14	≤10 n = 29	*p*	<90 n = 8	≥90 n = 35	*p*	>0.05 n = 11	≤0.05 n = 31	*p*	>0.5 n = 7	≤0.5 n = 35	*p*
Age (years)	9 (2–30)	12 (1–36)	0.483	10 (2–30)	12 (1–36)	0.577	9 (2–26)	12 (1–36)	0.502	6 (1–31)	12 (1–36)	0.229	12 (1–31)	10 (1–36)	0.774
Male sex	11 (73.3)	14 (50.0)	0.199	12 (85.7)	13 (44.8)	*0.019*	6 (75.0)	19 (54.3)	0.434	8 (72.7)	16 (51.6)	0.299	5 (71.4)	19 (54.3)	0.679
Type			0.709			0.373			0.333			0.183			>0.99
I	4 (26.7)	8 (28.6)		3 (21.4)	9 (31.0)		2 (25.0)	10 (28.6)		3 (27.3)	8 (25.8)		2 (28.6)	9 (25.7)	
II	7 (46.7)	8 (28.6)		7 (50.0)	8 (27.6)		5 (62.5)	10 (28.6)		6 (54.5)	9 (29.0)		4 (57.1)	11 (31.4)	
III	1 (6.7)	4 (14.3)		2 (14.3)	3 (10.3)		1 (12.5)	4 (11.4)		2 (18.2)	3 (9.7)		1 (14.3)	4 (11.4)	
IV	2 (13.3)	7 (25.0)		1 (7.1)	8 (27.6)		0 (0.0)	9 (25.7)		0 (0.0)	9 (29.0)		0 (0.0)	9 (25.7)	
VI	1 (6.7)	1 (3.6)		1 (7.1)	1 (3.4)		0 (0.0)	2 (5.7)		0 (0.0)	2 (6.5)		0 (0.0)	2 (5.7)	
Severe form	9 (60.0)	4 (14.3)	*0.004*	8 (57.1)	5 (17.2)	*0.013*	6 (75.0)	7 (20.0)	*0.006*	6 (54.5)	7 (22.6)	0.066	2 (28.6)	11 (31.4)	>0.99
Macrocephaly	9 (60.0)	5 (17.9)	*0.008*	8 (57.1)	6 (20.7)	*0.035*	5 (62.5)	9 (25.7)	0.089	6 (54.5)	7 (22.6)	0.066	2 (28.6)	11 (31.4)	>0.99
CI	10 (66.7)	8/27 (29.6)	*0.027*	10 (71.4)	8/28 (28.6)	*0.019*	6 (75.0)	12/34 (35.3)	0.056	6 (54.5)	11/30 (36.7)	0.476	4 (57.1)	13/34 (38.2)	0.421
eCSF protein	6/12 (50.0)	12/25 (48.0)	>0.99	5/12 (41.7)	13/25 (52.0)	0.728	3/7 (42.9)	15/30 (50.0)	>0.99	2 (18.2)	15/25 (60.0)	*0.031*	2/6 (33.3)	15/30 (50.0)	0.662
eCSF GAGs	8/12 (66.7)	13/25 (52.0)	0.491	9/12 (75.0)	12/25 (48.0)	0.166	5/7 (71.4)	16/30 (53.3)	0.674	8 (72.7)	13/25 (52.0)	0.295	4/6 (66.7)	17/30 (56.7)	>0.99
dPVS	14 (93.3)	15 (53.6)	*0.015*	14 (100.0)	15 (51.7)	*0.001*	7 (87.5)	22 (62.9)	0.240	11 (100.0)	17 (54.8)	*0.007*	7 (100.0)	21 (60.0)	0.075
eWML load	12/14 (85.7)	9 (32.1)	*0.003*	11/13 (84.6)	10 (34.5)	*0.006*	8 (100.0)	13/34 (38.2)	*0.003*	7 (63.6)	14 (45.2)	0.484	2 (28.6)	19 (54.3)	0.410
SCC	10 (66.7)	20 (71.4)	0.742	9 (64.3)	21 (72.4)	0.726	4 (50.0)	26 (74.3)	0.217	6 (54.5)	23 (74.2)	0.270	3 (42.9)	26 (74.3)	0.176

Data are presented as median (minimum–maximum) or counts (percentages)

Italic values indicate significance of *p* value (*p* < 0.05)

*p* statistical significance, *CSF* cerebrospinal fluid, *Severe form* Hurler syndrome (MPS I) and severe form of MPS II, *Macrocephaly* +2SD or 98%, *CI* cognitive impairment (score < 70), *eCSF protein* elevated cerebrospinal fluid protein (>32 mg/dl), *eCSF GAGs* elevated CSF glycosaminoglycans (>250 ng/ml), *dPVS* dilated perivascular spaces (number ≥ 10), *eWML load* elevated white matter lesion load (>0.4 cm^3^), *SCC* spinal cord compression (absence of CSF in any direction and/or myelomalacia)

The significance level was set at p < 0.05. Data were analysed using IBM-SPSS version 22.0.

## Results

Forty-three MPS patients performed clinical and developmental tests, CSF and neuroimaging studies over 3 years. The mean age of the patients was 13.7 years (age range 0.9–36 years). Severe forms of the disease (Hurler syndrome and severe form of MPS II) were observed in thirteen patients (30.2%). Macrocephaly (+2SD or 98%) was present in 32.6% of the patients. Based on IQ and development testing, 41.9% of the patients had cognitive impairment.

All the scores (Evans’ index, third ventricle width and callosal angle) used to assess the supratentorial ventricular enlargement in MPS patients presented a moderate correlation with the CSF aqueductal flow. Also, the ventricular CSF volume correlated with the CSF aqueductal flow, which did not occur with the total CSF volume and the subarachnoid CSF volume (Table 1). The third ventricle width showed a high inverse correlation with the brain volume (r_s_ = −0.61; p < 0.001) and Evans’ index showed the highest correlation with the ventricular CSF volume (r_s_ = 0.87; p < 0.001). In addition, Evans and third ventricle width scores had large correlations (r_s_ = 0.64 and r_s_ = 0.65, p < 0.001, respectively) with the total CSF volume. The other factor tested that correlated with the total CSF volume was the dilated PVS. The craniocervical junction stenosis significantly correlated with the cervical CSF flow (r_s_ = 0.46; p < 0.002) and was inversely correlated with the subarachnoid CSF volume.

The CSF opening pressure did not correlate either with the three measures of ventriculomegaly or with the ventricular CSF volume (Table 1). Also, no correlation was found between the CSF opening pressure and the CSF aqueductal flow (r_s_ = −0.23, p = 0.177), or the score for craniocervical stenosis (r_s_ = 0.19, p = 0.258). Table 1 also shows significant correlations between the dilated PVS and the WM lesion load with the ventricular CSF volume.

**Fig. 3:**
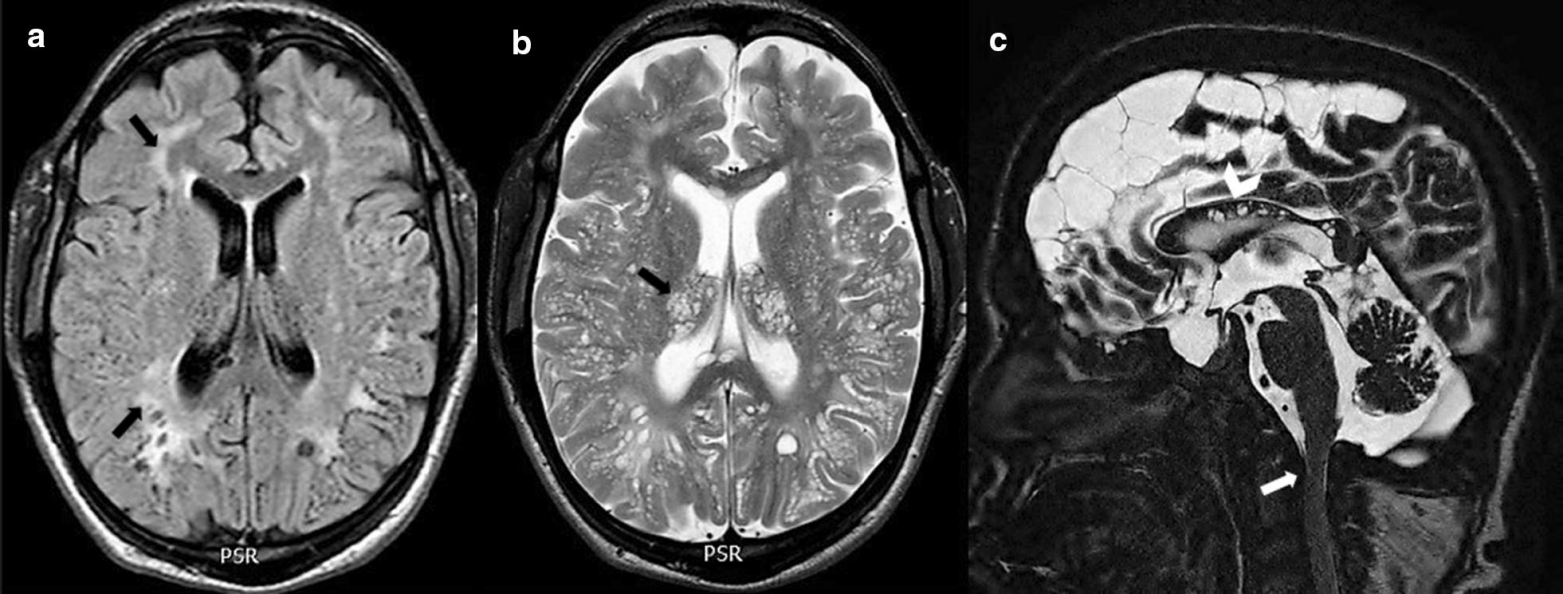
Brain MRI scans of a 20-year-old male patient affected by MPS II (attenuated form). Axial FLAIR image **a** shows periventricular and subcortical (arrows) white matter lesions (lesion load = 1.8%). Axial T2-weighted image **b** shows dilated perivascular spaces (score 3) prominently seen in the thalami and basal ganglia (arrow) and enlargement of subarachnoid spaces. Midsagittal T2-weighted image **c** shows dilated perivascular spaces within the corpus callosum (arrowhead), and effacement of CSF (arrow) and spinal stenosis at C1–C2 (score 1)

**Fig. 4:**
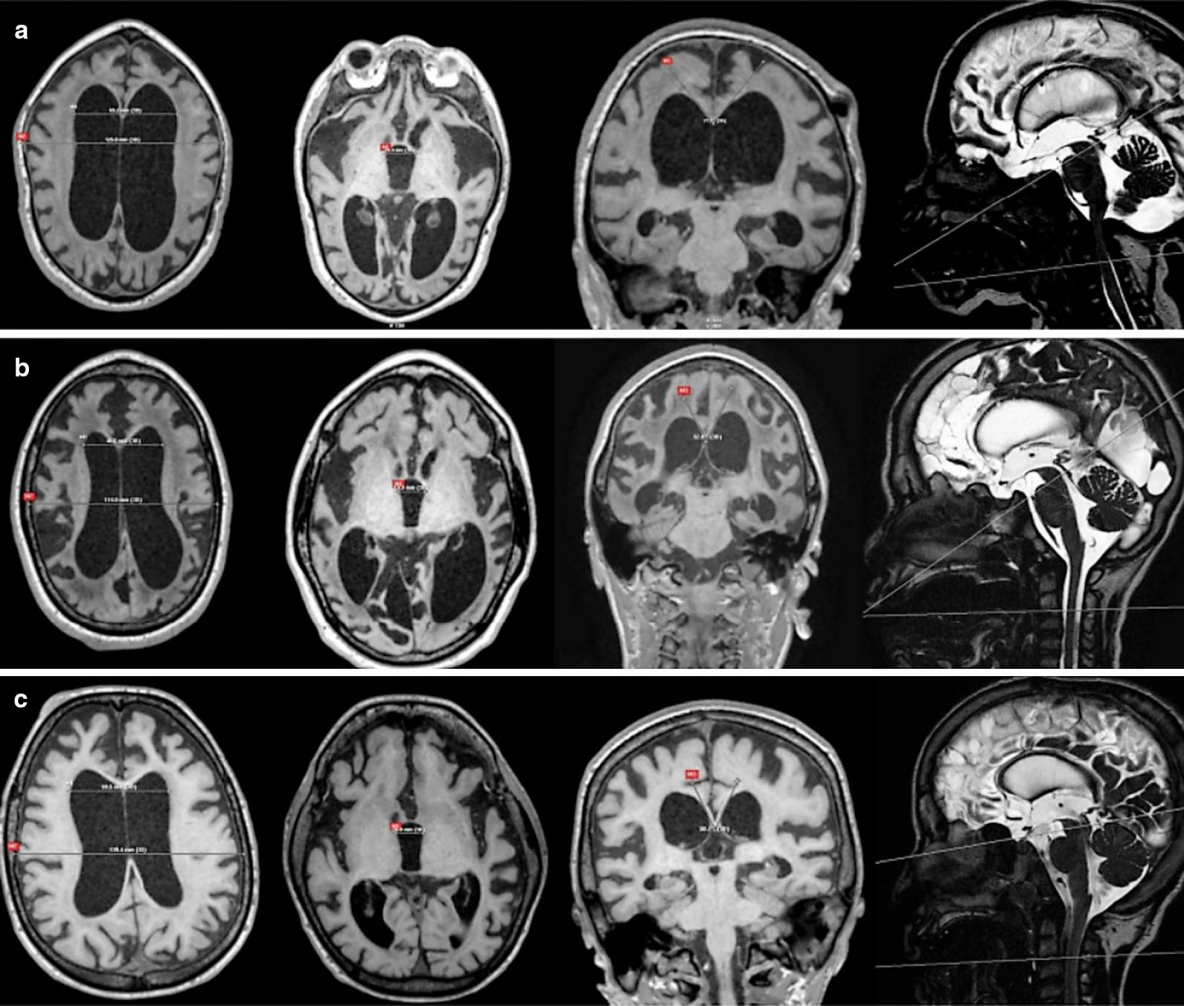
The three indices used for the assessment of ventricular size and the sagittal scout view sequences used for CSF flow quantifications at aqueductal level and C2–C3 level. **a** a 9-year-old girl with MPS I (Hurler syndrome): Evans’ index (EI) = 0.51. Width of III ventricle (WTV) = 19.4 mm. Callosal angle (CA) = 77.9°. CSF opening pressure (CSF OP) = 17.5 cm H_2_O. Aqueductal CSF stroke volume (ACSV) = 0.04 ml/CC. Cervical CSF stroke volume (CCSV) = 0 ml/CC; **b** a 10-year-old boy with MPS II (severe form): EI = 0.42. WTV = 14.9 mm. CA = 52.8°. CSF OP = 40 cm H_2_O. ACSV = 0.03 ml/CC. CCSV = 0.13 ml/CC; **c** a 8-year-old boy with MPS III A. EI = 0.43. WTV = 16.8 mm. Callosal angle = 50.7°. CSF opening pressure = 17.5 cm H_2_O. ACSV = 0.13 ml/CC. CCSV = 0.49 ml/CC

Table 2 shows the relationship between the clinical, CSF and neuroimaging features of MPS patients with ventriculomegaly and CSF flow. CSF GAG levels had no significant association with ventricle enlargement or CSF flow. The presence of ten or more dilated PVS showed significant association with ventriculomegaly, especially to the third ventricle width (Peto odds ratio 8.56, 95% CI 2.23–32.88), and also with elevated CSF stroke volume at the level of the cerebral aqueduct (Peto odds ratio 7.27, 95% CI 1.72–30.74). The WM lesion severity was significantly higher in patients with ventriculomegaly. The presence of craniocervical stenosis showed no significant association with decreased cervical CSF stroke volume. Representative cases are described in Figs. 3 and 4.

## Discussion

One of the biggest challenges with MPS patients is to distinguish communicating hydrocephalus from ventricular dilatation secondary to brain atrophy. Although conventional MRI sequences reveal morphological findings, when there is concern that developing hydrocephalus may require surgical management dedicated neuroimaging studies, including CSF flow measurement, may be indicated [15]. Ventricular dilation may be related to ventricular wall modifications induced by an increase in the pressure gradient between ventricular CSF and extraventricular CSF [32]. PC-MRI has the ability to measure ACSV, the elevation of which is associated with hydrocephalus. This is the first time that this technique has been described in MPS patients.

Evans’ index presented the highest correlation with ventricular CSF volumetrics and higher brain volume. Despite advances in brain imaging and volumetric analysis, this simple linear measurement continues to be fast, reliable and feasible for neurosurgical practice. The increased third ventricle width, which is a marker of brain atrophy in patients with multiple sclerosis [33, 34], might also be a surrogate marker of brain atrophy in MPS patients. The third ventricle divides the thalamic hemispheres, and thalamic atrophy may give rise to ex vacuo enlargement of the third ventricle. The measurement of the callosal angle, another supportive marker for the diagnosis of hydrocephalus and predictor of a positive outcome after shunting [35], presented good correlation with the other scores and proved to be useful. For this reason, we recommend the assessment of ventricular size by these three indices, which may enhance the diagnostic accuracy of hydrocephalus in MPS patients [36].

Aqueductal CSF stroke volume was significantly correlated with ventricular measurements and CSF ventricular volume. Chiang et al. demonstrated that the magnitude of ACSV is linked to the ventricular morphology [37], which is consistent with the finding by Poncelet et al. that the lateral compressive motion of the thalami on the third ventricle during the cardiac cycle modulates the CSF flow in the aqueduct [38]. Cerebrospinal fluid oscillations through the aqueduct appear to depend directly on CSF venting from the cranial cavity, resulting from both arterial inflow and the compliance of the craniospinal cavity [29]. In patients with communicating hydrocephalus, ventricular pulsations play a major role in cerebral pressure damping during vascular brain expansion, and ventricular dilation seems to be an adaptive response to changes in subarachnoid intracranial CSF pulsations [32]. With regard to patients with MPS, we believe that the obstruction of CSF reabsorption associated with cortical venous system hypertension due to impaired venous drainage caused by deformation of the skull base involve a reduction in the compliance of the subarachnoid space and limit total arterial pulsation toward the ventricles, increasing the ACSV.

The relationship between ventricle enlargement and decreased brain volume, and also the increased total CSF volume, which includes the CSF of subarachnoid spaces, might lead straight to the conclusion that ventriculomegaly is mainly due to cerebral atrophy. However, using the acquired knowledge from the patients with INPH who have also been found to have larger intracranial CSF volumes [39], it is more likely that, with decreased uptake of CSF by the arachnoidal granulations due to the deposition of storage material in the meninges, MPS patients might have developed a parallel pathway for CSF reabsorption, which could be the extracellular space of the brain. In accordance with this theory, we found a correlation between an increased number of dilated PVS with high CSF flow through the aqueduct, but also with larger total CSF volumes.

Cerebrospinal fluid opening pressure did not correlate with ventriculomegaly, CSF volume or CSF flow. In accordance with our results, the relationship between ICP, also considered as CSF opening pressure, and cerebral ventricle indices was shown as unreliable in pediatric and adult patients with communicating hydrocephalus and INPH [12–14]. Also, the assessment of changes in ventricular size gave no reliable prediction of changes in ICP [13]. The possible explanations are: (1) the development of ventriculomegaly may require a longer time period as the arachnoid granulations fail to maintain their baseline removal of CSF, secondary to deposition of GAGs in subarachnoid spaces [40], which may initially result in elevation of the mean ICP, but as time elapses, assuming the PVS to serve as lymphatics of the brain, the CSF reabsorption may improve; (2) the compensatory enlargement of cerebral ventricles and subarachnoid spaces in response to loss of brain tissue (hydrocephalus ex vacuo) in severe forms of MPS, may contribute to a reduction in ICP. In daily clinical practice changes in ventricular size assessed by neuroimaging often are used as predictors of changes in ICP. In this context, short-term and long-term changes should be taken into account.

In MPS patients, the WM lesion severity is associated with ventriculomegaly. The loss of myelin, axons and oligodendoglial cells causes the environment to become more hydrophilic [41]. However minimal it might be, this is certain to increase the resistance to movement of free water through the extracellular space of the brain. Thus, there will be a tendency for CSF to back up in the ventricles, adding another mechanism to the impaired pathways of CSF egress from the ventricles to the subarachnoid space [39]. Based on our results, ventriculomegaly is associated with a severe phenotype, increased cognitive decline and brain structural changes. Moreover, ventriculomegaly can be hypertensive or not. Thus, it is possible that atrophy, visualized as dilated cortical sulci and ventriculomegaly, may represent parenchymal involvement of the disease and also may be a partial sequela of a form of communicating hydrocephalus [2].

This study provides new information for a better understanding of ventriculomegaly in MPS patients, including its relationship with the elevated aqueductal CSF flow. It also reinforces the importance of considering the amount of dilated PVS as a biomarker for the balance between production and CSF reabsorption. Moreover, our study describes a possible temporal correlation of the clinical and neuroimaging findings with some of the histopathological events in the brain of MPS patients. Lee et al. proposed the natural course of cerebral involvement in MPS based on the MRI findings, and also postulated that cribriform changes occurred first, followed by WM changes and, last, atrophy [6]. Our study provides new data about the association of these changes with reabsorption failure of CSF in MPS patients. Besides that, we believe that the accumulation of CSF within the intracranial tissue is a major determinant of the clinical signs of hydrocephalus, more so than ventriculomegaly or elevated aqueductal CSF flow.

However, a weakness of the present study is the lack of correlation between ventriculomegaly and intra- and extracranial venous mean flow to test the hypothesis that venous hypertension due to reduced venous blood outflow may also play a role in the genesis of ventricular dilatation. Therefore, further studies are necessary to correlate cerebral venous blood flow with obstruction in cerebral veins, venous drainage anomalies, skull base abnormalities and communicating hydrocephalus in MPS patients. The T2 through-plane used for CSF volumetrics has low resolution. This is a technical limitation of our study instead of using T1-MPRAGE sequence which could increase the sensitivity of volumetric measurements comparison. Moreover, because ventricular size has limited specificity with regard to pathophysiology of hydrocephalus, it is very likely that a stand-alone measurement of ACSV has poor specificity for differential diagnosis, as previously noted in INPH and in predicting the response to shunt surgery [23]. In addition, as ACSV is highly machine- and technique-dependent, it is recommended to first perform CSF flow studies on a number of healthy patients without dilated ventricles to determine what is normal on that scanner. Then, when a MPS patient with suspected hydrocephalus is evaluated, a stroke volume at least twice that value would be required before recommending shunting [16]. Taking all of these factors together, it is our opinion that a combination of positive supplemental tests coupled with neurological deterioration can increase predictive accuracy in the diagnosis of hydrocephalus in MPS patients.

## Conclusions

Brain ventricular size and ventricular CSF volume had significant association with ACSV in MPS patients. CSF opening pressure (ICP) had no association with any of the above measurements. It is possible that MPS patients are more heavily reliant on reabsorption via the extracellular space of the brain. Perivascular spaces may represent the initial phase of abnormal CSF circulation, and ventriculomegaly may represent the later stages.

Although we have a better understanding of biomarkers associated with ventriculomegaly in MPS patients, these still do not provide a certain diagnosis for hydrocephalus or improve the accuracy of patient selection for surgical treatment. Concomitant analysis of venous and CSF flows using PC-MRI is necessary to search for impaired venous outflow and reduced intracranial compliance due to jugular foramina narrowing and retrograde venous hypertension.

## Abbreviations

MPS: mucopolysaccharidoses
GAGs: glycosaminoglycans
CSF: cerebrospinal fluid
PC: phase-contrast
MRI: magnetic resonance imaging
INPH: idiopathic normal pressure hydrocephalus
ICP: intracranial pressure
ACSV: aqueductal CSF stroke volume
VPS: ventriculoperitoneal shunting
PVS: perivascular spaces
WM: white matter
