# Correction to: Correlation of CSF flow using phase-contrast MRI with ventriculomegaly and CSF opening pressure in mucopolysaccharidoses

**DOI:** 10.1186/s12987-017-0076-z

**Published:** 2017-10-13

**Authors:** Amauri Dalla Corte, Carolina F. M. de Souza, Maurício Anés, Fabio K. Maeda, Armelle Lokossou, Leonardo M. Vedolin, Maria Gabriela Longo, Monica M. Ferreira, Solanger G. P. Perrone, Olivier Balédent, Roberto Giugliani

**Affiliations:** 1 Post-Graduate Program in Medical Sciences, Universidade Federal do Rio Grande do Sul, Porto Alegre, Brazil; 2 Medical Genetics Service, Hospital de Clínicas de Porto Alegre, Rua Ramiro Barcelos 2350, Porto Alegre, RS 90035‑903 Brazil; 3 Medical Physics and Radioprotection Service, Hospital de Clínicas de Porto Alegre, Porto Alegre, Brazil; 4 Clinical Engineering, Santa Casa de Misericórdia de Porto Alegre, Porto Alegre, Brazil; 5 Image Processing Unit, Amiens University Hospital, Amiens, France; 6 Department of Neuroradiology, DASA Group, São Paulo, Brazil; 7 Department of Radiology, Massachusetts General Hospital, Boston, USA; 8 Anesthesiology Service, Hospital de Clínicas de Porto Alegre, Porto Alegre, Brazil

## Correction to: Fluids Barriers CNS (2017) 14:23 DOI 10.1186/s12987-017-0073-2

After publication of the article [1], it has been brought to our attention that the full funding acknowledgement is missing from the original article. It should read as below.

**Acknowledgements**

We gratefully acknowledge the financial support provided by the Fundo de Incentivo à Pesquisa e Eventos of Hospital de Clínicas de Porto Alegre for the publication of the present study.

**Funding**

The publication fees were covered by the Fundo de Incentivo à Pesquisa e Eventos of Hospital de Clínicas de Porto Alegre.
